# Improved Framework for Tractography Reconstruction of the Optic Radiation

**DOI:** 10.1371/journal.pone.0137064

**Published:** 2015-09-16

**Authors:** Eloy Martínez-Heras, Federico Varriano, Vesna Prčkovska, Carlos Laredo, Magí Andorrà, Elena H. Martínez-Lapiscina, Anna Calvo, Erika Lampert, Pablo Villoslada, Albert Saiz, Alberto Prats-Galino, Sara Llufriu

**Affiliations:** 1 Center of Neuroimmunology, Service of Neurology, Hospital Clinic and Institut *d*′Investigacions Biomèdiques August Pi I Sunyer (IDIBAPS), Barcelona, Spain; 2 Laboratory of Surgical NeuroAnatomy (LSNA). Facultat de Medicina. Universitat de Barcelona, Barcelona, Spain; 3 Comprehensive Stroke Center, Department of Neuroscience. Hospital Clinic and Institut *d*′Investigacions Biomèdiques August Pi I Sunyer (IDIBAPS), Barcelona, Spain; 4 Medical Imaging Platform, Institut *d*′Investigacions Biomèdiques August Pi I Sunyer (IDIBAPS), Barcelona, Spain; University of Minnesota, UNITED STATES

## Abstract

The optic radiation (OR) is one of the major components of the visual system and a key structure at risk in white matter diseases such as multiple sclerosis (MS). However, it is challenging to perform track reconstruction of the OR using diffusion MRI due to a sharp change of direction in the Meyer’*s* loop and the presence of kissing and crossing fibers along the pathway. As such, we aimed to provide a highly precise and reproducible framework for tracking the OR from thalamic and visual cortex masks. The framework combined the generation of probabilistic streamlines by high order fiber orientation distributions estimated with constrained spherical deconvolution and an automatic post-processing based on anatomical exclusion criteria (AEC) to compensate for the presence of anatomically implausible streamlines. Specifically, those ending in the contralateral hemisphere, cerebrospinal fluid or grey matter outside the visual cortex were automatically excluded. We applied the framework to two distinct high angular resolution diffusion-weighted imaging (HARDI) acquisition protocols on one cohort, comprised of ten healthy volunteers and five MS patients. The OR was successfully delineated in both HARDI acquisitions in the healthy volunteers and MS patients. Quantitative evaluation of the OR position was done by comparing the results with histological reference data. Compared with histological mask, the OR reconstruction into a template (OR-TCT) was highly precise (percentage of voxels within the OR-TCT correctly defined as OR), ranging from 0.71 to 0.83. The sensitivity (percentage of voxels in histological reference mask correctly defined as OR in OR-TCT) ranged from 0.65 to 0.81 and the accuracy (measured by F1 score) was 0.73 to 0.77 in healthy volunteers. When AEC was not applied the precision and accuracy decreased. The absolute agreement between both HARDI datasets measured by the intraclass correlation coefficient was 0.73. This improved framework allowed us to reconstruct the OR with high reliability and accuracy independently of the acquisition parameters. Moreover, the reconstruction was possible even in the presence of tissue damage due to MS. This framework could also be applied to other tracts with complex configuration.

## Introduction

Diffusion-weighted imaging (DWI) is a magnetic resonance imaging (MRI) modality that measures the amount and directionality of water molecule diffusion within tissue [1]. From DWI, the direction of maximal diffusivity along axonal fibers can be estimated for each voxel. This information enables the reconstruction of the architectural configuration of white matter (WM) trajectories between regions of interest (ROIs) through fiber tractography [2]. Detailed tractography studies have created virtual anatomic atlases of WM connections in the human brain [3, 4] that agree with results from dissection and tracer studies [5, 6]. However, fiber tracking has several limitations that may affect the reliability and reproducibility of outcomes resulting from different approaches. First, the image resolution in diffusion MRI (dMRI) is usually several orders of magnitude higher than the actual axons size, so each voxel contains information from hundreds of thousands of axon fibers [7]. Moreover, the presence of crossing fibers, highly curved or diverging fiber bundles, might affect the directionality of each fiber bundle within the voxel [8]. The selection and positioning of the specific seed ROIs used to perform the tractography may lead to considerable variability in the results [9]. In addition, several tractography algorithms are available; the two main distinguishing factors relate to how WM fiber tracts are modeled within a voxel and how the tracts are reconstructed [10]. Deterministic streamline tractography is primarily based upon streamline algorithms in which the local tract direction is defined by the major eigenvector of the diffusion tensor and can produce anatomically faithful reconstructions of WM fascicules. However, in general, branching will not be represented and this approach may be less effective in regions where considerable fiber crossing is present and curvature is high [11]. Probabilistic tractography algorithms based on a fiber orientation density function provide information about the confidence that one can assign to a reconstructed trajectory and can indicate the occurrence of branching [12]. The high-order tractography model, constrained spherical deconvolution (CSD), is advisable for performing a robust tractography [13]. Deterministic tractography can also be estimated from the fiber orientation distribution (FOD) and obtain robust tracking in the presence of crossing fibers. However, deterministic tractography with high order models is prone to missing existing streamlines [14]. The use of high order integration over fiber orientation distributions (iFOD2) [15] derived from CSD could improve tracking in regions containing complex fiber architecture [16]. Unfortunately, probabilistic fiber tracking frequently generates anatomically implausible streamlines. New methods based on anatomical information from whole-brain tractography, such as anatomically-constrained tractography (ACT) [17], have been developed to provide a more biologically accurate reconstruction through dynamic thresholding strategy. However, this method does not resolve the problem of false positives and systematic errors in the tractography. For that reason, we propose the use of post-processing based on anatomical exclusion criteria (AEC) to define the final tracking results and exclude aberrant streamlines.

The optic radiation (OR) is one of the major components of the human visual system. It links the lateral geniculate nucleus (LGN) with the visual cortex and is one of the most difficult tracts to reconstruct with dMRI-based methods [18] due to a sharp change of direction in the Meyer’*s* loop and the presence of kissing and crossing fibers along the pathway [19]. The topography of the OR is evident in histological sections [20], however, the reconstruction of the OR in vivo suffers from inaccuracy and different levels of success have been reported [21, 22]. Variability in the results may derive from a lack of consistency in the selection of the seed regions and tractography algorithms (deterministic and probabilistic) used for fiber tractography [23, 24] as well as in the selection or exclusion of streamlines representing the OR [25]. To reconstruct the OR through tractography some authors have placed a waypoint seed distal to the Meyer’*s* loop in order to map the rapidly curving anterior fibers [26–29] while few studies have been able to obtain the OR from LGN seeds [19, 30, 31]. Although most techniques succeeded in representing the simplest anterior-posterior streamlines of the OR, aberrant fibers were eliminated by visual inspection [32] or by looking at the anatomical plausibility of the highest scoring pathways [33]. These approaches can induce biases in the position, shape, size and length of the streamline distribution [34] and do not ensure the representation of the most reliable pathway based on neuroanatomical knowledge.

The OR can be damaged in diseases such as multiple sclerosis (MS). The visual pathway can be used to evaluate the interplay of different mechanisms of damage in MS such as inflammation (lesions and normal appearing WM), demyelination and axonal damage and also particular mechanisms such as trans-synaptic degeneration [35–38]. The presence of lesions with low fractional anisotropy can erroneously terminate the tracking algorithm from conventional DTI-tractography or cause a deviation of the streamlines at the level of the lesions [39]. As such, an accurate and reproducible reconstruction of the OR and other tracts is essential to evaluate the consequences of WM damage in MS or other neurological diseases, and for surgery planning [24].

The purpose of this article is to introduce an improved tractography framework to reconstruct the OR using the thalamus and visual cortex as seed and target masks that combines probabilistic streamline fiber tracking by iFOD2 and automatic post-processing based on AEC. We aim to demonstrate its precision, accuracy and reproducibility in two different single-shell high angular resolution diffusion-weighted imaging (HARDI) datasets from healthy volunteers and explore its applicability in the presence of tissue damage caused by MS.

## Materials and Methods

### Material and Imaging

Ten healthy volunteers and five patients with MS were recruited for this study. The included healthy volunteers [3 male and 7 female, mean (SD) age 30 (± 8) years] did not have any known neurological or psychiatric diseases. The MS patients [2 male and 3 female, mean (SD) age 35 (± 9) years] were recruited from the MS Unit at the Hospital Clinic in Barcelona. The Ethics Committee of Hospital Clinic of Barcelona approved the study and a signed consent form was obtained from all the participants.

### MRI data acquisition

MRI images were acquired on a 3T Magnetom Trio (Siemens, Erlangen, Germany) scanner, using a thirty-two channel phased-array head coil. One 3D T1-weighted structural and two different DWI sequences were acquired in the same scanning session. The 3D-structural image was a T1-weighted MPRAGE sequence with the following acquisition parameters: TR: 2050 ms, TE: 2.41 ms TI: 1050 ms, flip angle: 9^°^, 192 contiguous sagittal slices with 0.86 x 0.86 x 0.9 *mm* ^3^ voxel size, 256 x 256 matrix size. Two HARDI datasets were acquired, HARDI A: TR/TE, 16600/110 ms; acquisition matrix, 154 x 154; 100 contiguous axial slices; 1.5 mm isotropic voxel size; b value, 1500 s/*mm* ^2^; 16 minutes acquisition time and HARDI B: TR/TE, 6200/84 ms; acquisition matrix, 96 x 96; 55 contiguous axial slices; 2.5 mm isotropic voxel size; b value, 1000 s/*mm* ^2^; 4 minutes acquisition time. Both HARDI datasets used the same 60 numbers of gradient directions [40] and a single baseline image acquired at 0 s/*mm* ^2^. Parallel imaging was applied with a geometric reduction factor of 2 to reduce the distortion caused by susceptibility differences at tissue interfaces. In addition, a 2D field map sequence was acquired to correct geometric distortions of the DWI caused by susceptibility differences between air-bone or air-tissue interfaces. The field map was obtained at two different echo times (*TE*_1_ = 4.92 ms and *TE*_2_ = 7.38 ms) and in the same slice prescription, slice thickness and field of view as both HARDI datasets.

### Construction and inspection of seed and target masks

The 3D-structural image served as an anatomical landmark to obtain GM ROIs in each hemisphere. The parcellation scheme from Freesurfer (FS) software [41] was used as a first approximation to outline the thalamus and visual cortex that were going to serve as the seed and target masks, respectively. In patients, the 3D-structural image was also used to manually create a lesion mask with the ITK-SNAP toolkit [42]. Afterwards, an automated lesion filling was applied to the 3D-structural image to improve segmentation and registration steps in patients [43]. The seed and target masks were then further inspected and corrected by an experienced neuroanatomist using AMIRA 5.1 software (Mercury Computer Systems, Berlin, Germany) to reduce inaccuracies and limitations of the parcellation algorithm. The thalamus seed mask used in this study corresponds to the Thalamus-Proper parcellation in the Desikan-Killiany Atlas while the visual cortex target mask was obtained by merging the pericalcarine, cuneus, lateraloccipital, lingual and precuneus parcellations. As the LGN was not properly parcellated in the thalamus-proper ROI, we manually selected and included this nucleus in the seed mask.

### Proposed framework to reconstruct the OR

**Fig 1:**
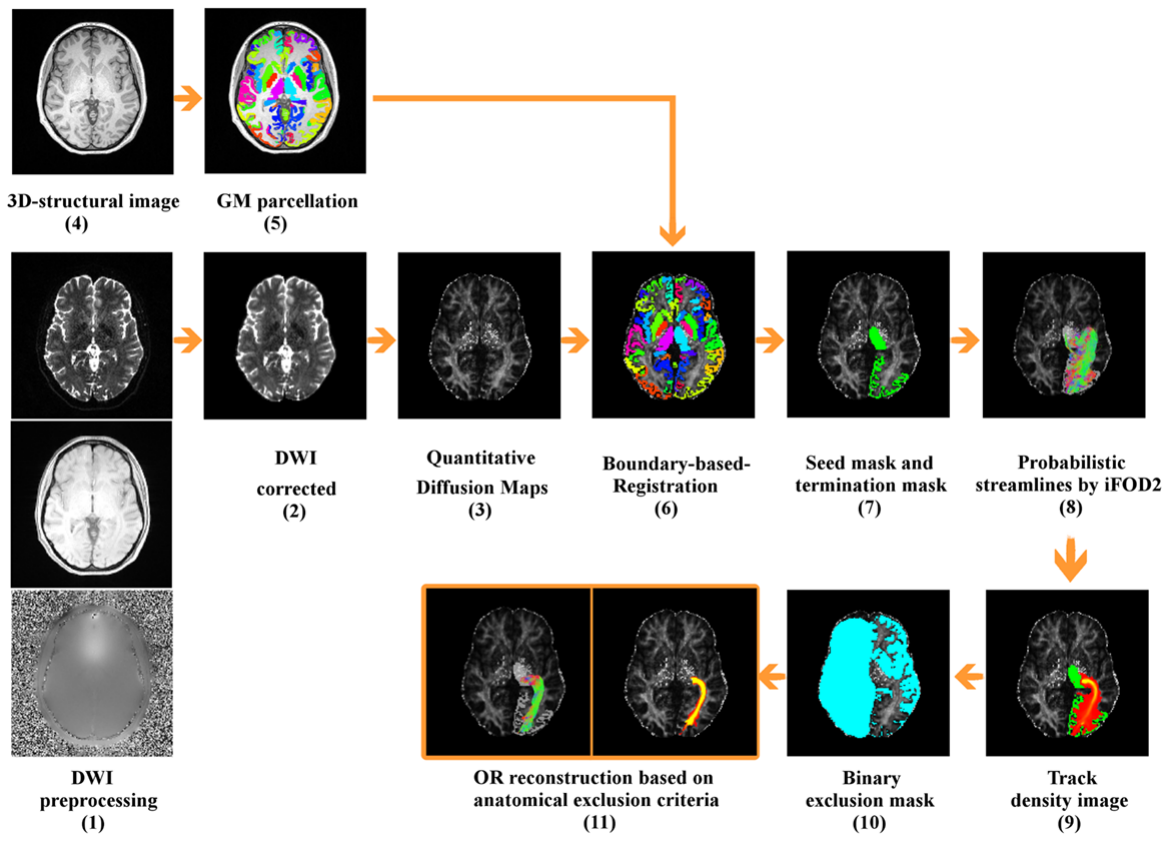
Tractography reconstruction framework of the optic radiations. (1) Standard preprocessing of the DWIs including Echo Planar Imaging distortion correction, eddy current distortion correction and head motion correction. (2) Distortion correction of the DWI. (3) Quantitative diffusion fractional anisotropy (FA) mapping. (4-5) Subcortical segmentation and cortical parcellation from FS of the 3D-structural image. (6) Registration of the structural images to the corresponding DWI sequence. (7) Seed and target masks. (8) Probabilistic streamline fiber tracking by high order integration over fiber orientation distributions (iFOD2) derived from constrained spherical deconvolution (CSD) with a maximum harmonic order of 8 and use of ACT during tracking. (9) Conversion of the tract file into a track density image. (10) Exclusion mask comprising CSF, whole contralateral hemisphere and ipsilateral GM regions. (11) Final optic radiation reconstruction in track density image and 3D tract file.

The schematic diagram of the framework is presented in Fig 1. Initially, we performed standard preprocessing of the DWIs that included geometric distortion correction for echo planar images (EPI) with fieldmap images and head motion correction. Fieldmap-based unwarping of the EPI was done using PRELUDE to unwrap the phase and FUGUE to compute the distortion by means of FMRIB Software Library (FSL, http://www.fmrib.ox.ac.uk/fsl) [44]. After applying eddy current corrections we rotated the gradient vectors in both HARDI datasets in order to compensate for head motion. This was followed by the registration of the structural images to the corresponding HARDI dataset using the Boundary-Based-Registration method [45]. Probabilistic tractography from seed to target masks was performed in each hemisphere using the MRtrix3 package (https://github.com/jdtournier/mrtrix3) [46]. A set of 100,000 streamlines was generated from the seed mask in only one direction and stopped as soon as it entered the target mask. The default step size, curvature and FOD amplitude threshold (0.1) were used. We applied probabilistic streamline fiber tracking by iFOD2 derived from CSD with a maximum harmonic order of 8 [47, 48], and used FreeSurfer tissue segmentations for ACT. Anatomically unrealistic streamlines were present in the final tracking results. To correct for this problem, we automatically applied AEC to the results. We first converted the tract file into a track density image at native resolution based on the map of the fraction of tracks that enter each voxel. We set a threshold of 1% of the maximum value included in the track density image [32, 49] and selected the biggest cluster in order to get a temporary tract. The temporary tract file retained plausible streamlines that connected the seed mask to the target mask and removed streamlines of low confidence, i.e. false positives. Second, we applied a binary exclusion mask to the temporary tract file comprised of the cerebrospinal fluid (CSF), whole contralateral hemisphere and ipsilateral GM regions that did not include the seed and target masks. As a result, any streamline crossing the midline or reaching cortical and subcortical GM regions outside the seed and target masks were excluded. Next, we converted the tract file back into a track density image and the resulting images were scaled to a range of [0–1] [50] to enable the comparison between individual samples. In patients with MS we overlaid the lesion mask obtained with ITKsnap on the reconstructed OR to identify how many patients presented lesions at that level.

In order to compare our framework with conventional DTI-tractography, we performed the same pre-processing and applied deterministic fiber tracking based on the fiber assignment by continuous tracking (FACT) [51] method in the healthy volunteers with the same seed and target mask. This fiber tracking was done with MRtrix3 software using the default setting parameters and an FA threshold of 0.1.

### Qualitative and quantitative evaluation of the method

Visual inspection of the OR resulting from streamlines in both HARDI datasets was performed by a neuroanatomy specialist to evaluate concordance in morphology and position with the known anatomical description of this pathway [52]. Quantitative evaluation of the OR position was done by comparing the results with histological reference data of the OR [20, 53]. The OR obtained in each HARDI dataset was converted into a template (tractography-constructed template, OR-TCT) (S1 File). To do so, each individual track density image was aligned to the highest resolution sequence (HARDI A) and was normalized into a standardized space (Montreal Neurological Institute, MNI152) and then averaged. The histological reference data was edited to exclude GM and CSF regions from the MNI152 structural template image in order to preserve only WM regions. It was then converted to a binary mask to incorporate the information of all the subjects included in the histological atlas. The sensitivity, precision, specificity and F-measure of our method were calculated in each HARDI dataset to assess the similarities between the OR-TCT and the histological reference data.

Sensitivity was measured as the proportion of voxels within the reference mask that were correctly defined as OR in the OR-TCT.

$$\begin{array}{c} Sensitivity=\frac{True\;positives}{\left(True\;positives+False\;negatives\right)} \end{array}$$ (1)

Specificity was the proportion of voxels outside the reference mask that were correctly classified as non-OR voxels in the OR-TCT.

$$\begin{array}{c} Specificity=\frac{True\;negatives}{\left(True\;negatives+False\;positives\right)} \end{array}$$ (2)

Precision was calculated as the proportion of voxels within the OR-TCT that were correctly defined as OR.

$$\begin{array}{c} Precision=\frac{True\;positives}{\left(True\;positives+False\;positives\right)} \end{array}$$ (3)

We included the F1 score, a measure of the tests accuracy, in order to estimate the weighted harmonic mean of precision and sensitivity.

$$\begin{array}{c} F_{1}=2\cdot \frac{Precision\cdot Sensitivity}{\left(Precision+Sensitivity\right)} \end{array}$$ (4)

We also performed a quantitative evaluation of the OR in the absence of the AEC. In order to evaluate the reproducibility of the OR reconstruction we compared the results obtained from the two different HARDI datasets in each subject. The agreement in OR volume was evaluated with Bland-Altman plots [54] and the intraclass correlation coefficient (ICC). Considering the well-recognized anatomical differences between left hemisphere (lh) and right hemisphere (rh) [55], we merged comparison from lh and rh into the same analysis. We used SPSS v.18 to construct the Bland-Altman plots and calculate the ICC.

## Results

**Fig 2:**
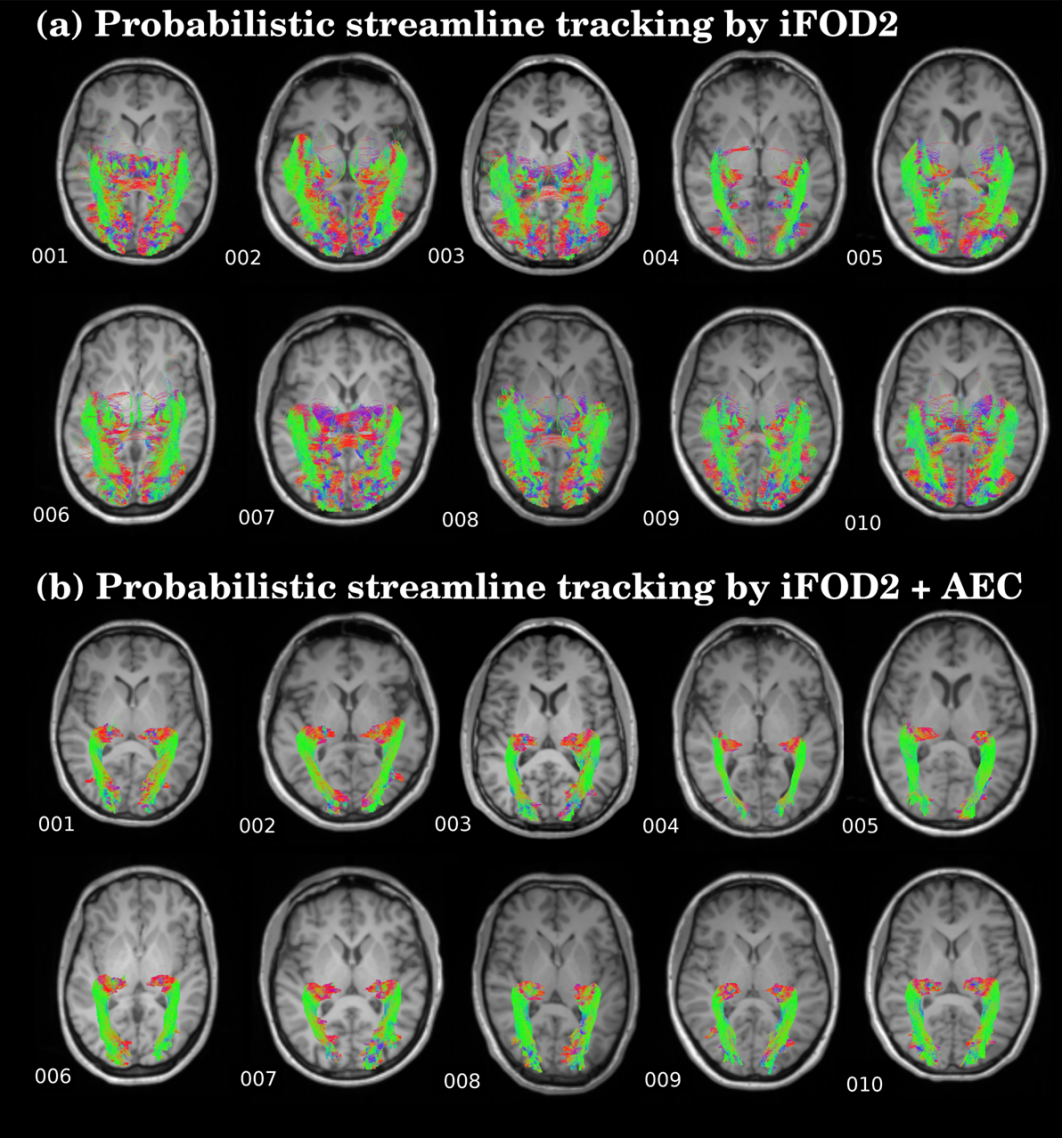
Streamlines of the reconstructed OR in ten healthy subjects: (a) Probabilistic streamlines fiber tracking by iFOD2. (b) Probabilistic streamlines fiber tracking by high order integration over fiber orientation distributions (iFOD2) adding the anatomical exclusion criteria (AEC).

**Fig 3:**
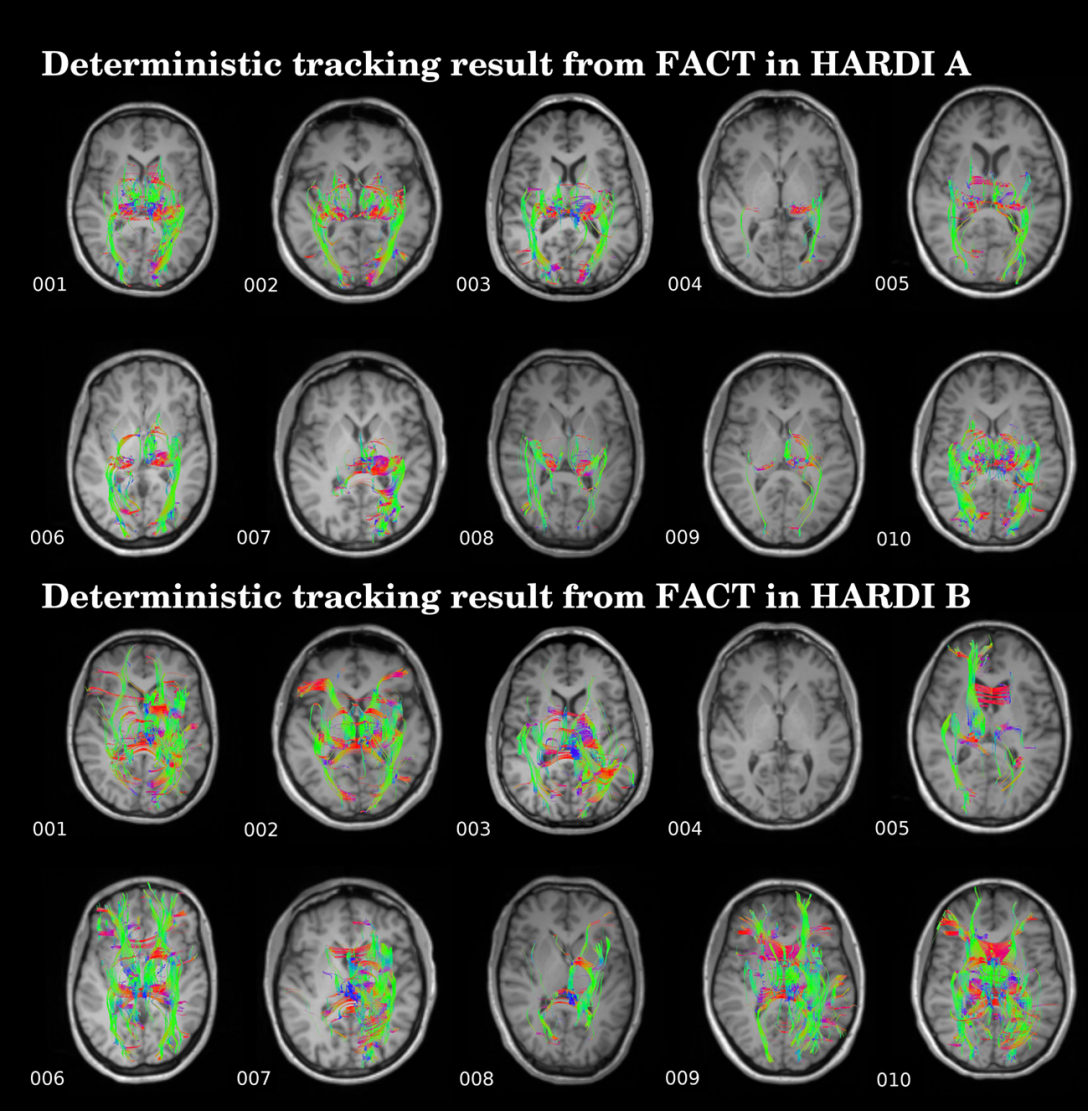
Deterministic DTI tractography based on fiber assignement by continuous tracking (FACT) in ten healthy subjects in two HARDI datasets.

The proposed framework reconstructed OR fiber tracking in both HARDI datasets in all the healthy volunteers included in the study. The results were visually concordant with the anatomical description of the streamlines (Fig 2b). The resulting fiber trajectories emerged from the posterior part of the thalamus, including the LGN. A portion of the streamlines looped anterior and laterally around the temporal horn of the lateral ventricle forming the Meyer’*s* loop, where it sharply turned in a posterior direction, while another portion followed a more direct trajectory. The streamlines then followed the curvature of the ventricular atrium and reached the visual cortex. On the contrary, the application of conventional DTI-tractography was unable to solve the complex configuration in the Meyer’*s* loop, generated unrealistic streamlines and in some cases was unable to even produce an OR reconstruction in two subjects (see Fig 3). Morever, the results were largely dependent upon the sequence used, with worse reconstruction obtained with HARDI B.

**Fig 4:**
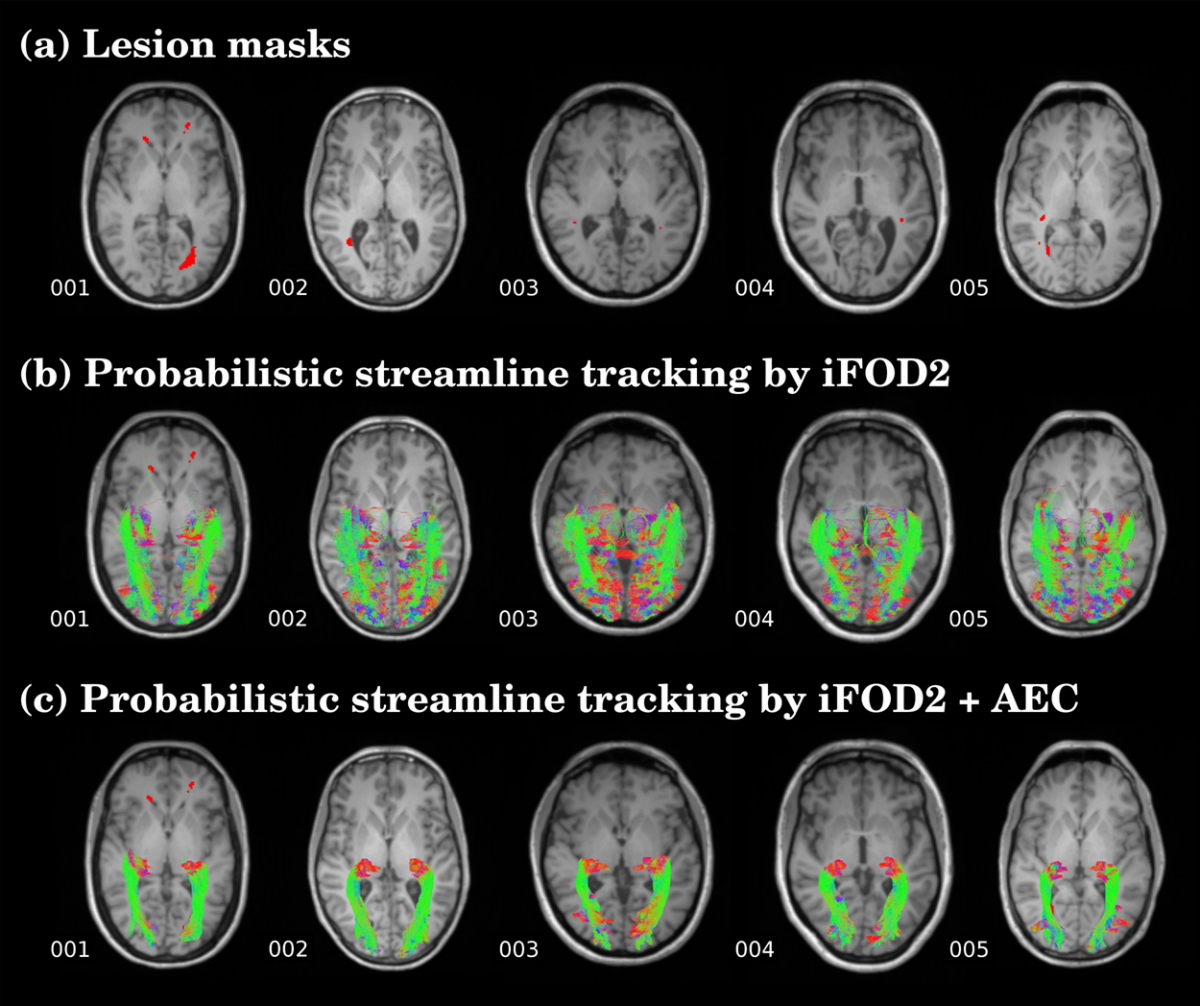
Streamlines of the reconstructed OR in five patients with multiple sclerosis: (a) Lesion masks is shown in red. (b) Probabilistic streamlines fiber tracking by iFOD2. (c) Probabilistic streamlines fiber tracking by high order integration over fiber orientation distributions (iFOD2) adding the anatomical exclusion criteria (AEC).

In patients with MS, the OR were also accurately reconstructed with the proposed framework (Fig 4c) although all the subjects presented lesions within the tracts (Fig 4a).

**Fig 5:**
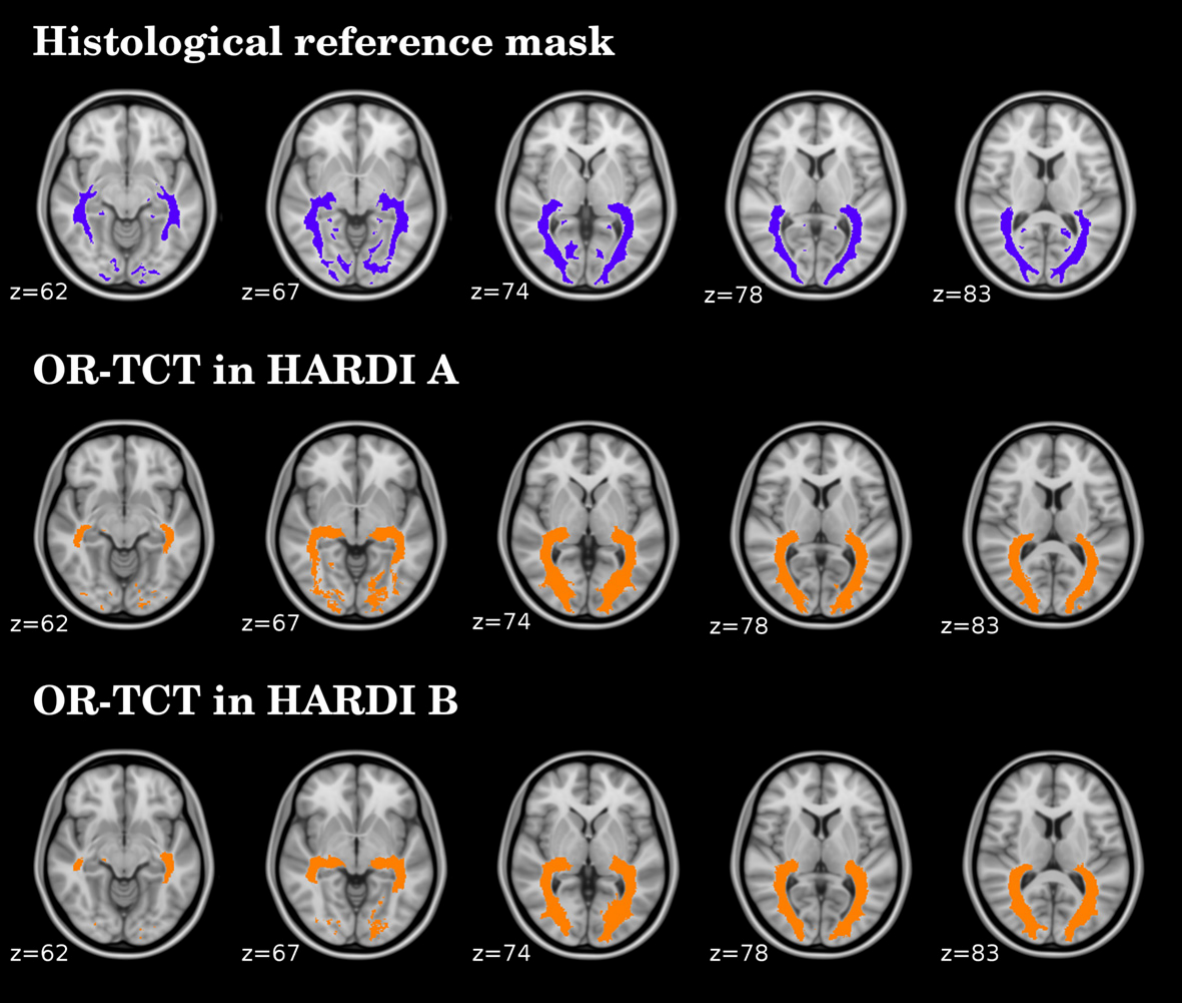
Anatomical correspondence between the histological reference mask (in blue color) and tractography-constructed templates (OR-TCT) from both HARDI datasets (in orange color).

The framework provided good anatomical correspondence between the OR-TCT and the histological reference data in healthy volunteers (see Fig 5). The sensitivity, precision, specificity and F-measure results are shown in Table 1. The results demonstrate a good match between the OR-TCT in both HARDI datasets and the histological reference mask. Precision ranged from 0.71 to 0.83 depending on the HARDI dataset or hemisphere explored (false positive from 17 to 29%, while accuracy ranged from 0.73 to 0.76. In the HARDI A dataset, with smaller voxel size, the sensitivity was lower than HARDI B while the precision was higher (Table 1). The application of AEC also increased the precision and accuracy ranges in MS patients (S1 Table).

**Table 1:** Comparison between tractography results and histological reference data in healthy volunteers.

		OR-TCT (iFOD2)		OR-TCT (iFOD2 + AEC)		reference mask^a^
		HARDI A^b^	HARDI B^c^	HARDI A^b^	HARDI B^c^	Histological
Tract volume (*cm* ^3^), mean (±*SD*)	lh	44.87 (±7.69)	52.38 (±14.46)	12.25 (±2.73)	16.81 (±4.89)	18.4 (±2.1)
	rh	41.69 (±8.00)	43.37 (±11.03)	12.01 (±2.03)	14.99 (±3.52)	18.4 (±1.3)
Sensitivity	lh	0.99	0.99	0.71	0.81	-
	rh	0.99	1.00	0.65	0.70	-
Precision	lh	0.18	0.14	0.83	0.71	-
	rh	0.20	0.17	0.83	0.79	-
Specificity	lh	0.92	0.90	1.00	0.99	-
	rh	0.93	0.92	1.00	1.00	-
F-measure	lh	0.30	0.25	0.77	0.76	-
	rh	0.33	0.29	0.73	0.74	-

^a^Volumes in the histological reference mask were obtained from Clatworthy et al., 2010.

^b^HARDI A: 1.5 mm isotropic voxel size; b-value, 1500 s/*mm* ^2^

^c^HARDI B: 2.5 mm isotropic voxel size; b-value, 1000 s/*mm* ^2^

Abbreviations:

AEC: automatic post-processing based on anatomical exclusion criteria.

iFOD: high order integration over fiber orientation distributions.

lh: left hemisphere.

OR-TCT: optic radiation tractography-constructed template.

rh: right hemisphere.

When AEC was not applied, streamlines not concordant with prior anatomical knowledge appeared in all subjects (Figs 2a and 4b) and the accuracy of the OR compared to the histological mask decreased (range from 0.25 to 0.33) while sensitivity was very high (range from 0.99 to 1.0) due to the overestimation of WM streamlines with false positives (Table 1); false positive in non-AEC ranged from 80 to 86% (S2 Table).

**Fig 6:**
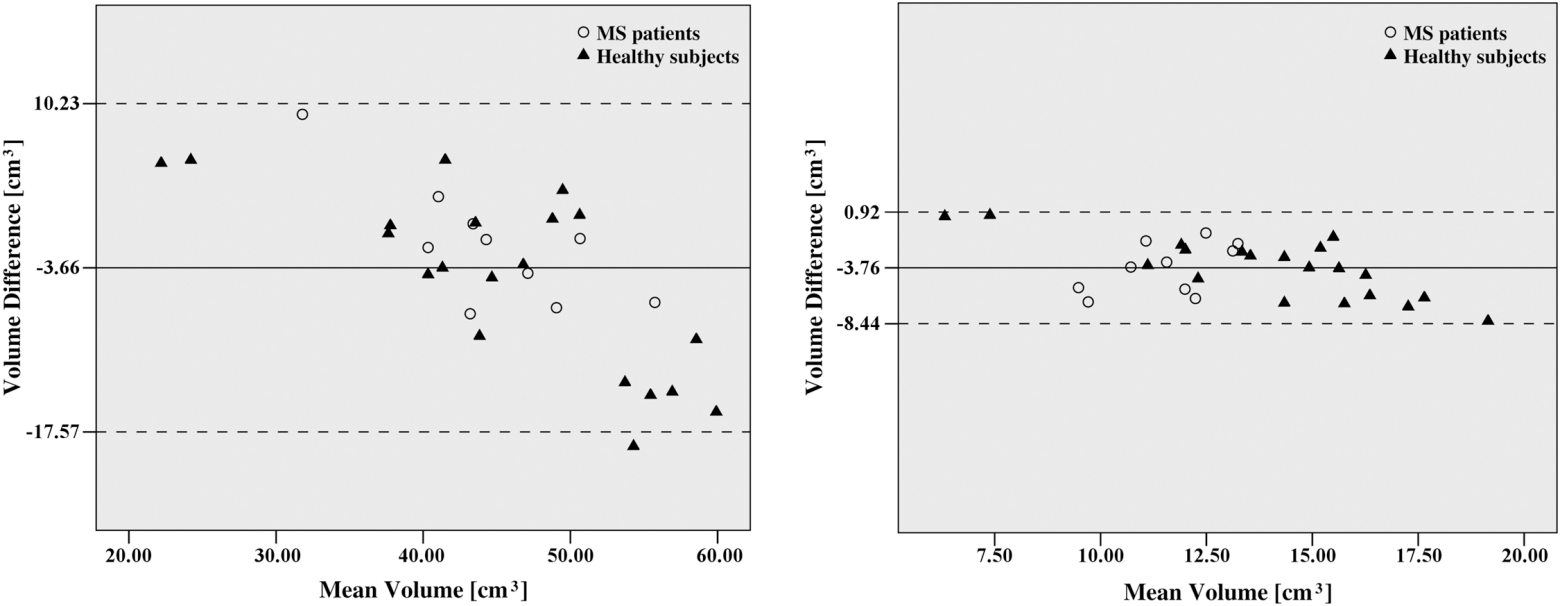
Bland-Altman plots comparing the mean volume of OR in both HARDI datasets. Left panel corresponds to non-AEC and right panel corresponds to results with AEC method. The volume of optic radiation in each subject is the mean of both hemispheres. Most observed differences between the OR volumes in the two sequences are within mean ± 1.96 SD. Middle line indicate mean differences and dashed lines are limits of agreement, defined as mean difference plus (upper line) and minus (lower line) 1.96 SD of differences.

Bland-Altman plots showed good agreement between the OR tracking obtained in both HARDI datasets for all subjects (Fig 6). We did not detect any outliers, suggesting that the technique is reproducible. The OR was bigger in HARDI B, where voxel size is larger, than in HARDI A (Table 1) but we did not detect a systematic bias between both images. The ICC measuring the absolute agreement was 0.73 (95% confidence interval: 0.50 to 0.86, p < 0.001).

## Discussion

We present an improved tractography framework that combines the use of available software packages to reconstruct the OR from thalamus and visual cortex masks with the automatic exclusion of aberrant streamlines through anatomical criteria. Contrary to conventional DTI-tractography this methodology enabled the reconstruction of the whole OR in high concordance with anatomical knowledge and high accuracy in comparison with the histological reference data. In patients with MS, the OR was obtained in all cases even though there were lesions in these areas. Furthermore, this technique was applied in two different HARDI datasets and the resulting OR fiber tracks were comparable, suggesting good reliability of the approach. Our framework used high order probabilistic streamlines derived from CSD and automatic post-processing based on AEC. The AEC steps included the application of a threshold and the selection of the maximally connected volume to retain plausible OR reconstructed streamlines that connect the seed to the target mask. Most widely used methods are based on the application of a threshold to eliminate low probability connections not corresponding to the OR. Another method, ACT, uses information from high-resolution images to decrease the presence of anatomically implausible streamlines generated on fiber tracking. Despite the application of ACT we observed a large number of false positives streamlines projecting to areas outside the visual cortex (Fig 2a). The use of the top 1% track density threshold in AEC steps was found to be the most convenient to preserve sensitivity and precision by largely reducing false positives voxels (from more than 60% to 17%) rather than increase false negatives (from 3% to 40%) (S3 Table). Besides, we applied exclusion masks based on anatomical criteria in order to exclude non OR tracks. This was done by excluding streamlines ending in other GM regions apart from the seeds and target masks, as well as CSF and contralateral hemisphere voxels, that did not match with the classical description of the OR anatomy. With the application of AEC steps the accuracy of the streamlines improved in both healthy volunteers and MS patients.

We were able to reconstruct the whole OR, including the Meyer’*s* loop, using only a seed and a target masks that corresponded to the initial and the final part of the tract (the thalamus and the visual cortex, respectively). In comparison with previous reports, we did not require the use of waypoints in the Meyer’*s* loop to reconstruct the part of the tract with higher curvature. The decision to use the whole thalamus as a seed region was made in light of experimental evidence showing that the visual pathway in primates not only connects the LGN with the calcarine cortex but also includes some projections from the pulvinar nuclei in the posterior thalamus [56]. We manually delineated the thalamus to include the LGN, which tended to be systematically missing with FS segmentation [57]. The visual cortex target mask was built by taking the original primary visual cortex FS parcellation (pericalcarine) and merging the surrounding visual association areas (cuneus, lateraloccipital, lingual and precuneus) in order to ensure the inclusion of extrastriate projections of the OR described in humans [58].

To validate the anatomical correspondence of our results in healthy subjects, we created a template of our OR (OR-TCT) and compared it with a histological reference mask, which serves as a gold standard. The comparison of the OR-TCT with histological reference data shows the biological reliability of the tractography framework but also presents some drawbacks that limit the comparison: the histological reference data is based on myelin-stained histological serial sections of 10 human brains and includes the LGNs and the striate GM areas, increasing the size of the template. Since we assume that meaningful tracts only propagate through WM, we excluded GM regions from the histological reference mask when calculating the precision and sensitivity. As such, the differences in precision and sensitivity between both techniques arise not only from the limitation of the tractography algorithm but also from the effect of unavoidable distortions specific to fixation, cutting, staining and mounting of the histological samples. Nevertheless, the accuracy of the results in both HARDI datasets suggested that our OR-TCT matched well with the histological reference data in location and extent (see Fig 5).

Our study has several strengths. First, the proposed framework was used in different sequences with similar results, demonstrating good reproducibility. We compared the volume of the OR in two HARDI datasets that differ in voxel size and b-value. The results were consistent in both sequences although they presented differences in terms of absolute volume, with higher volumes in the sequence with larger voxel size. Those differences can be related to variations in partial volume effects, contrast in the angular domain and signal-to-noise ratio derived from the different spatial resolutions and b-values [59]. Second, the OR was reconstructed in all MS patients, even in the presence of lesions and microstructural damage. Although the number of patients included was low, the technique appears to be capable of generating a reliable reconstruction of the OR. This can be very useful in the evaluation of the consequences of global and local damage and could be applied to other neurological diseases or for neurosurgical procedures.

However, there are some limitations in the proposed methodology inherent to the technique. First of all, the method is dependent on the seed and target masks positioning, which is the only operator-dependent step and can influence the tractography results. In order to decrease the probability of bias, the ROIs should be properly segmented to obtain reliable and reproducible positioning. In our study, the automatic segmentation from FS was not suitable so we decided to correct the seed and target masks manually to ensure an accurate reconstruction based on neuroanatomical knowledge. However, our framework is intended to be used with any selected seed and target masks. Second, this methodology was only evaluated in specific HARDI datasets; therefore, it would be of interest to assess the reliability of OR tracking in higher resolution sequences such as multi-shell HARDI and diffusion spectrum imaging (DSI). Third, the inclusion of the AEC method reduces the sensitivity. This is due in part to exclusion of the most anterior extent of the Meyer’*s* loop streamlines where fibers projecting to the putamen and temporal cortex, not corresponding to the OR, have been described [60]. Despite the decrease in sensitivity, the AEC method increased the precision and accuracy, obtaining an OR reconstruction similar to the histological atlas. Finally, we didn’t analyze the presence of damage of the OR in MS patients because our aim was to introduce a reliable tractography methodology. Further clinical studies with higher number of subjects allowing the analysis of changes in MS patients and longitudinal evaluations to assess the reproducibility of the technique are advisable.

## Conclusion

We present a framework for tractography reconstruction that was able to reconstruct the OR with high accuracy and reliability by combining high order probabilistic fiber tracking derived from CSD and automatic post-processing based on anatomical exclusion criteria. Moreover, this technique was applied to different HARDI datasets with equivalent results. Finally, despite the presence of WM damage, it was possible to reconstruct the OR in patients with MS. This framework could be used to accurately represent other tracts with complex configuration and in pathologies affecting the WM.

## Supporting Information

S1 File OR-TCT. Optic radiation tractography-constructed template in both HARDI datasets. The OR obtained in each HARDI dataset was converted into a template. To do so, each individual track density image was aligned to the highest resolution sequence (HARDI A) and was normalized into MNI152 space at 1mm. The “S1 File” is composed by the OR reconstruction in both HARDI datasets in binarized masks.
(ZIP)

S1 Table Comparison between tractography results and histological reference data in MS patients. (PDF)

S2 Table Comparison between the application or not of the AEC method. (PDF)

S3 Table Comparison between the application or not of the top 1% track density threshold in AEC steps. (PDF)
