# Supplementary material for: Improved Framework for Tractography Reconstruction of the Optic Radiation
Source: PLoS One. 2015 Sep 16;10(9):e0137064. doi: 10.1371/journal.pone.0137064 (PMC4573981; doi:10.1371/journal.pone.0137064)
Supplement: S1 Table — (PDF) [file pone.0137064.s002.pdf]

		OR-TCT (iFOD2)		OR-TCT (iFOD2 + AEC)		reference mask[a]
		HARDI A[b]	HARDI B[c]	HARDI A[b]	HARDI B[c]	Histological
Tract volume (cm^3), mean ($\pm SD$)	lh	45.83 (±5.41)	48.13 (±7.83)	10.00 (±2.42)	13.60 (±0.81)	18.4 (±2.1)
	rh	41.69 (±3.68)	42.99 (±9.53)	9.39 (±1.91)	13.26 (±1.53)	18.4 (±1.3)
Sensitivity	lh	0.98	0.98	0.66	0.60	-
	rh	0.98	0.98	0.56	0.57	-
Precision	lh	0.21	0.20	0.62	0.54	-
	rh	0.24	0.22	0.60	0.57	-
Specificity	lh	0.94	0.93	0.99	0.99	-
	rh	0.94	0.94	0.99	0.99	-
F-measure	lh	0.35	0.33	0.64	0.57	-
	rh	0.38	0.35	0.58	0.55	-

[a]Volumes in the histological reference mask were obtained from Clatworthy et al., 2010.

[b]HARDI A: 1.5 mm isotropic voxel size; b-value, 1500 s/mm^2

[c]HARDI B: 2.5 mm isotropic voxel size; b-value, 1000 s/mm^2

Abbreviations:

AEC: automatic post-processing based on anatomical exclusion criteria.

iFOD: high order integration over fiber orientation distributions.

lh: left hemisphere.

OR-TCT: optic radiation tractography-constructed template.

rh: right hemisphere.
