# Supplementary material for: Improved Framework for Tractography Reconstruction of the Optic Radiation
Source: PLoS One. 2015 Sep 16;10(9):e0137064. doi: 10.1371/journal.pone.0137064 (PMC4573981; doi:10.1371/journal.pone.0137064)
Supplement: S2 Table — (PDF) [file pone.0137064.s003.pdf]

		OR-TCT (iFOD2)		OR-TCT (iFOD2 + AEC)	
		HARDI A[a]	HARDI B [b]	HARDI A[a]	HARDI B[b]
False positive (%)	lh	82.22	85.57	16.69	28.95
	rh	80.13	82.70	17.21	21.22
False negative (%)	lh	0.11	0.11	34.25	16.64
	rh	0.12	0.08	44.51	33.19

[a]HARDI A: 1.5 mm isotropic voxel size; b-value, 1500 s/mm^2

[b]HARDI B: 2.5 mm isotropic voxel size; b-value, 1000 s/mm^2

Abbreviations:

AEC: automatic post-processing based on anatomical exclusion criteria.

iFOD: high order integration over fiber orientation distributions.

lh: left hemisphere.

OR-TCT: optic radiation tractography-constructed template.

rh: right hemisphere.
