# Supplementary material for: Improved Framework for Tractography Reconstruction of the Optic Radiation
Source: PLoS One. 2015 Sep 16;10(9):e0137064. doi: 10.1371/journal.pone.0137064 (PMC4573981; doi:10.1371/journal.pone.0137064)
Supplement: S3 Table — (PDF) [file pone.0137064.s004.pdf]

	FP	TP	FN	TN	VOL	SENS	PREC	SPEC	F-MEASURE
OR-TCT + AEC without threshold (lh)	55479 (66.72%)	27667	2461 (2.9%)	1737370	83146	0.92	0.33	0.97	0.49
OR-TCT + AEC without threshold (rh)	50823 (63.76%)	28881	2780 (3.5%)	1740493	79704	0.91	0.36	0.97	0.52
OR-TCT + AEC 1% threshold (lh)	4278 (16.7%)	21350	8778 (34%)	1805218	25628	0.71	0.83	1	0.77
OR-TCT + AEC 1% threshold (rh)	4279 (17.2%)	20591	11070 (44%)	1803684	24870	0.65	0.83	1	0.73

Number of voxels and percentage (%) are shown.

Abbreviations:

AEC: automatic post-processing based on anatomical exclusion criteria.

FN: False negatives.

FP: False positives.

lh: left hemisphere.

OR-TCT: optic radiation tractography-constructed template.

rh: right hemisphere.

TN: True negatives.

TP: True positives.
